# Livelihood Capital Effects on Famers’ Strategy Choices in Flood-Prone Areas—A Study in Rural China

**DOI:** 10.3390/ijerph19127535

**Published:** 2022-06-20

**Authors:** Yibin Ao, Ling Tan, Qiqi Feng, Liyao Tan, Hongfu Li, Yan Wang, Tong Wang, Yunfeng Chen

**Affiliations:** 1 College of Environment and Civil Engineering, Chengdu University of Technology, Chengdu 610059, China; aoyibin10@mail.cdut.edu.cn (Y.A.); tanling@stu.cdut.edu.cn (L.T.); fengqiqi@stu.cdut.edu.cn (Q.F.); tanliyao@stu.cdut.edu.cn (L.T.); lhf158084887012022@163.com (H.L.); 2 Department of Engineering Management, Sichuan College of Architectural Technology, Deyang 618014, China; 3 Faculty of Architecture and the Built Environment, Delft University of Technology, 2628 CD Delft, The Netherlands; 4 School of Construction Management Technology, Purdue Polytechnic Institute, Purdue University, West Lafayette, IN 47907, USA; chen428@purdue.edu

**Keywords:** farmers’ household, flood disaster, capital, livelihood strategy

## Abstract

The global climate change has resulted in huge flood damages, which seriously hinders the sustainable development of rural economy and society and causes famers’ livelihood problems. In flood-prone areas, it is imperative to actively study short and long-term strategies and solve farmers’ livelihood problems accordingly. Following the sustainable development analysis framework proposed by the Department for International Development (DFID), this study collects empirical data of 360 rural households in six sample villages in the Jialing River Basin of Sichuan Province, China through a village-to-household field questionnaire and applies the Multinominal Logit Model (MNL) to explore the influence of farmer households’ capital on livelihood strategy choice. Research results show that: (1) In human capital category, the education level of the household head has a significant positive impact on the livelihood strategies of farmers’ families; (2) In physical capital category, farmer households with larger space have more funds to choose among flood adaptation strategies; (3) In natural capital category, house location and the sale of family property for cash have the greatest negative impact on farmers’ livelihood strategies; (4) Rural households with more credit opportunities in financial capital are more willing to obtain emergency relief funds; (5) Farmers’ families helped by the village for a long time will probably not choose to move to avoid floods, but are more likely to choose buying flood insurance. This study provides an empirical reference for effective short and long term prevention and mitigation strategies design and application in rural in flood-prone areas.

## 1. Introduction

According to the International Emergency Disaster Database (EM-DAT), floods rank the first among all natural disasters in terms of frequency, total population, area and direct economic losses. China is one of the countries with the highest flood frequency and flood range in the world, and the economic losses caused by floods are extremely high. According to the statistical distribution of flood in China from 2000 to 2015, Sichuan is a region that suffers from floods frequently. Mianyang, Dazhou and Nanchong in Jialing River Basin have a wide range of exposure to flood disasters, and all of them have suffered from severe flood disasters. Dazhou suffered the “9.3” catastrophic flood in 2004, which directly caused an economic loss of 6.1 billion yuan. From July to August, 2020, Nanchong suffered a severe flood, resulting in a direct economic loss of 755 million yuan. In 2020, Mianyang suffered the biggest rainstorm and flood since the founding of the People’s Republic of China. The intensity, duration and severity of the process are rare in history. The continuous torrential rain caused frequent flood hazards in Mianyang, resulting in a direct economic loss of 2.968 billion yuan. Rural areas in China face more flood disasters and farmer households are the most basic disaster-bearing units. The sudden flood disasters have shown that the lack of flood disaster knowledge and capital reserves of farmers have become important issues. Choosing appropriate livelihood strategies during the disasters and in the long-term adaptation will greatly improve resilience and reduce famers’ vulnerability to future disasters. 

Following the sustainable development analysis framework of the Department for International Development (DFID), this study obtained the research data of 360 farmer households in six sample villages in Jialing River Basin through the questionnaire survey in villages and households, and used the Multinominal Logit Model (MNL) to explore the influence mechanism of livelihood capital on farmer households’ livelihood strategies in flood-prone areas step by step. The structure of the rest of this paper is as follows: Section 2 lists the literature results and Section 3 introduces the research area and research methods, and Section 4 analyzes the empirical results. Section 5 presents the conclusions and suggestions.

## 2. Literature Review

The literature review has revealed that the most critical factor to alleviate livelihood stress and improve resilience to disasters of farm households is to improve the livelihood capital of farm households [1]. From the perspective of livelihood capital, human, physical and social capital all have an impact on farmers’ changing livelihood strategies in the face of climate extremes [2]. Livelihood strategies are not only central to sustainable livelihoods, but can also provide important guidance for addressing the problems caused by disasters [3].

Cai [4] executed a quantitative study on the influencing factors of the restoration and reconstruction of farmers’ livelihood system in four poverty-stricken counties in Wenchuan areas, and the results show that the imbalance in the sub-capital of the livelihood capital of farm households is extremely high, resulting in the low livelihood of farm households in natural disasters. In the southwest minority areas, Zhuang et al. [5] find that due to the increased frequency of natural disasters, the incidence of poverty among low-income farmers increased, and most of the poor were trapped in the poverty afterwards. Cao et al. [6] analyze the coupling model and coordination degree of ecological vulnerability and economic poverty in 14 contiguous areas with special difficulties, and confirmed that the ecological vulnerability and economic poverty in the region coexist, and attention should be paid to the protection of the ecological environment. Yang et al. [7] analyze the financial policies implemented in various regions aiming in helping different types of farmers to get rid of poverty, and there are significant differences in scalability and sustainability of such policies and financial poverty alleviation has an important impact on the livelihood development of poor farmers.

Regarding livelihood capital influencing factors, Ma and Liu [8] use a multi-class logistic regression model to quantitatively simulate the relationship between farmers’ livelihood capital and livelihood strategies in Qingpu District, Shanghai under the background of rapid urbanization in China, and find that the average annual income of farmers is the key factor affecting the choice of farmers’ livelihood strategies. It is suggested to help farmers optimize their livelihood strategies and develop sustainable livelihoods. Yang [9] takes the desertification land closed to protection zones as the object, and empirically analyzes the relationship between farmers’ livelihood capital and livelihood strategies using the logistic regression model. The results show that farmers with much financial and social capital choose a variety of livelihood strategies. Su et al. [10] take the livelihood capital of farmers in Ganzhou District, Zhangye City as the research object to study the relationship between farmers’ livelihood capital and their livelihood strategies, and find that farmers with more natural capital tend to choose agricultural livelihood strategies, while the ones with more financial capital prefer off-farm livelihood strategies. Zhao [11] conducts a regression analysis on the relationship between the livelihood capital and livelihood strategies before and after the relocation of rural households for poverty alleviation. The results show that the relocation change the livelihood capital of farmers, and the livelihood strategies gradually tend towards non-agricultural livelihood strategies.

Furthermore, the livelihood strategies of residents will be changed constantly according to the adjustments of policies, institutions, external environment and personal livelihood capital [12]. For example, when rural residents face risks or impacts from natural disasters, famine, or ecological degradation, they often change their livelihood strategies according to their own capital [13]. There has been much research on livelihood strategies and their drivers in the context of disasters. Mentamo and Geda’s [14] analysis showed that the education level of the head of the household, access to credit, participation in a food for work programme and the land size owned by households were the key predictors of livelihood diversification. Rahut et al. [15] analyzed strategies for diversifying rural livelihoods and their impacts on household welfare, pointing out the importance of diversifying livelihoods to reduce poverty and increase household income. Hriday Lal Koirala et al. used PRA method to conduct field research and quantitative analysis of the livelihood capital status of farmers in The Merramzi Basin, the impact of livelihood capital on livelihood strategies and the sensitivity characteristics of different livelihood strategies to livelihood capitals [16]. 

It can been seen that existing research is limited in the sense that they focus on the relationship between livelihood capital and general livelihood strategies [17]. And they only examine several types of livelihood capital, and do not subdivide the impact of farmers’ household livelihood capital indicators on the choice of livelihood strategies for farmers [18]. Furthermore, the choices made to cope with short-term disaster and adapt to natural disasters after being hit by natural disasters in the long run are not separated [19]. Therefore, it is very necessary to study the relationship between livelihood capital and livelihood strategies in more detail with a systematic approach [20].

## 3. Methodology

### 3.1. Questionnaire Design

In this study, 360 rural households in six sample villages with frequent flood disasters in the Jialing River Basin of Sichuan province were interviewed to conduct empirical research using questionnaires. The main purpose is two-folds: the choice of livelihood strategies of households in flood-prone areas and the specific livelihood capital indicators of households in the sample area. 

(1) Independent variable: The selection of livelihood capital indicators is based on the principles of professionalism, experience, authenticity, feasibility and representativeness, according to the five-dimension criteria of livelihood capital proposed by the Department for International Development (DFID). These variables are divided into Human capital, Natural capital, Financial capital, Social capital and Physical capital on the first level. Furthermore, based on the context of the research area and referring to the domestic and foreign literature [17,18,19,20,21,22,23,24,25,26,27,28], sub-indicators of each first-level indicators are identified and categorized (see Table 1).

It is found that the age of the head of household, family size, illness status and education level will affect the family’s choice of disaster mitigation strategies, and will also have a significant impact on vulnerability factors such as sensitivity and adaptability. The family building structure is selected as the index of material capital, because it is of great significance to maintain building safety and family development [17]. Most of the damages and losses caused by flood impact occur in the housing structure, and the age of housing increases the vulnerability of flood impact [21]. Studies have also shown that families with a relatively high number of income members may have diversified income combinations [22]. High income and income diversification can also improve families’ ability to cope with and recover from floods, and reduce their social vulnerability to floods [21]. Credit opportunities provide uninsured families with a means to manage disaster losses, but after a serious incident, access to credit may be fragile [23]. Flood prevention is affected by community awareness and land use area. When the elements of the family at risk are exposed to disasters, the more times they are exposed, the more vulnerable they are to the power and influence of disasters, and the more vulnerable they are [24]. Therefore, the distance between houses and rivers is taken as the index of natural capital. In the event of a disaster, trusting neighbors and communities has a significant impact on reducing the experience of disaster conflicts [25]. Those families who have received recovery assistance from neighbors, stronger personal networks and higher levels of social capital recover faster [26]. Similarly, in terms of social capital, the public’s trust in the management organization after being severely damaged by major disasters will also affect the public’s preparedness for disasters [27].

When there are many independent variables in the practical problems studied, there may be a certain degree of correlation between two or more independent variables, which is called multicollinearity. If the collinearity trend of independent variables is obvious, the fitting effect of the model will be seriously affected [28,29,30]. In this study, the Variance inflation factor (VIF) is used to test multicollinearity. When the VIF value is greater than 10, it is considered that the variables have strong multicollinearity, which is unacceptable [31]. The VIF values of the independent variables in this research are all less than 3, indicating that there is no multicollinearity among the independent variables. The test results are shown in Table 1.

**Table 1:** Independent variable index, source and likelihood ratio test.

First- Level Indicator	Secondary Indicators	Indicator Meaning	Indicator Source	Collinearity Test	
				Tolerance	VIF
H Human capital	H1 Age of household head	0.2 = 18 years and under; 0.6 = 18 to 30 years old; 1 = 31 to 50 years old; 0.8 = 51 to 60 years old; 0.4 = 61 years and over.	[32]	0.997	1.003
	H2 Education level household head	0.2 = Ll literacy; 0.4 = Primary school; 0.6 = Junior high school; 0.8 = High school and secondary school; 1 = University and above	[33]	0.997	1.003
	H3 Family illness	1 = Yes; 0 = No	[34]	0.997	1.003
	H4 Total family size	1 = Less than two people; 0.8 = Two to four people; 0.6 = Four to six people; 0.4 = Six to eight people; 0.2 = Eight or more people	[32]	0.998	1.002
P Physical capital	P1 House area	0.25 = Less than 100 square meters; 0.5 = 100 to 150 square meters; 0.75 = 150 to 200 square meters; 1 = More than 200 square meters	[32]	0.953	1.050
	P2 House age	1 = Less than 10 years; 0.8 = 10 to 20 years; 0.6 = 20 to 30 years; 0.4 = 30 to 40 years; 0.2 = More than 40 years	[24]	0.992	1.008
	P3 House structure	1 = Reinforced concrete; 0.8 = Brick concrete; 0.6 = Cob house; 0.4 = wooden house; 0.2 = thatched cottage	[35]	0.972	1.029
	P4 Household livestock value	0.2 = Blow 1 thousand yuan; 0.4 = 1 to 2 thousand yuan; 0.6 = 2 to 3 thousand yuan; 0.8 = 3 to 4 thousand yuan; 1 = 4 thousand yuan and above	[36]	0.978	1.022
	P5 Value of household items	0.2 = Blow 10 thousand yuan; 0.4 = 10 to 50 thousand yuan; 0.6 = 50–100 thousand yuan; 0.8 = 100–150 thousand yuan; 1 = 15 thousand yuan and above	[36]	0.960	1.042
N Natural capital	N1 Own land area	0.2 = 0 to 1 mu; 0.4 = 1 to 2 mu; 0.6 = 2 to 3 mu; 0.8 = 3 to 4 mu; 1 = 4 mu and above	[24]	0.974	1.027
	N2 Family location (the distance between the house and the river)	0.2 = Below 0.5 km; 0.4 = 0.5 to 1 km; 0.6 = 1 to 1.5 km; 0.8 = 1.5 to 2 km; 1 = More than 2 km	[37]	1.000	1.000
	N3 Drain condition	1 = Yes; 0 = No	[38]	0.974	1.027
F Financial capital	F1 Number of households with income	0.2 = One person; 0.4 = Two people; 0.6 = Three people; 0.8 = Four people; 1 = Five or more people	[39]	0.923	1.083
	F2 Average annual household income	0.2 = Blow 10 thousand yuan; 0.4 = 10 to 20 thousand yuan; 0.6 = 20 to 30 thousand yuan; 0.8 = 30 to 40 thousand yuan; 1 = 40 thousand yuan and more	[40]	0.772	1.295
	F3 Credit opportunity	1 = Yes; 0 = No	[35]	0.926	1.080
	F4 Borrowing opportunity	1 = Yes; 0 = No	[35]	0.930	1.075
S Social capital	S1 Community help during disasters	1 = Yes; 0 = No	[32]	0.462	2.163
	S2 Helped by neighbors during disasters	1 = Yes; 0 = No	[41]	0.622	1.608
	S3 Trust in village managers	1 = Trust all; 0.75 = Mostly trust; 0.5 = Half trust; 0.25 = Few trust; 0 = Hardly trust	[35]	0.490	2.039
	S4 Occupation in the village group	1 = Yes; 0 = No	[42]	0.669	1.495

Dependent variable: The farmer’s family can choose short-term and long-term livelihood strategies. During the flood, the short-term livelihood strategies of farmers’ families include: relying on government and social organizations’ relief funds, borrowing money from banks or loan companies, selling livestock for cash, using past savings, borrowing money from relatives and friends, and taking temporary jobs in nearby areas that are not affected by the disaster to earn money. According to the existing literature and the characteristics of rural residents in Sichuan, this study sums up six farmers’ families’ choice of livelihood strategies for flood response in short-term regarding their livelihood capitals [43]. The choices of long-term livelihood adaptation strategies of farmers’ families in flood-prone areas include: increasing the height of houses, improving drainage ditches, participating in flood emergency training, purchasing flood insurance, changing the type and date of crop planting, choosing to move, and increasing agricultural irrigation measures. In this study, seven farmers’ families’ livelihood capital choices of livelihood strategies for flood adaptation are selected.

In short-term flood coping strategies, after the disaster, first of all, farmers’ families will have no financial income, and some families will use their accumulated savings to ensure their daily expenses and recover the losses caused by the flood disaster [32]. The government and social welfare organizations will often provide materials and financial assistance to the disaster areas [44], so as to help farmers get through the flood difficulties as soon as possible. Farmers can also rely on this source of funds to relieve the pressure of temporary lack of financial income due to the flood. Floods have a great impact on farmers’ families, and will cause great harm to their houses and crops. When the past savings can’t support the family’s recovery, the head of household who is short of funds will choose to borrow some funds from relatives or friends whose families are relatively rich or haven’t been hit by floods to quickly recover the losses caused by floods [32], or immediately borrow money from banks and loan companies as a response measure to tide over the floods [45]. Severe flood impacts often force farmers to sell livestock to maintain their basic consumption. Even wealthy farmers have to reduce livestock to cope with floods [46]. When floods occur, farmers sell livestock to reduce their feed expenses, so as to obtain cash. And it is an important risk treatment mechanism to earn money by taking temporary jobs in nearby areas that are not affected by disasters [45]. Floods reduce farmers’ agricultural labor, and the labor force is idle. Choosing to stay nearby without being affected by floods can make up for the loss of crop income caused by floods, thus protecting family income.

In the long-term flood adaptation strategy, because of the perennial flooding, families will raise the foundation of newly repaired houses appropriately according to the situation of previous floods [20], so as to avoid flooding the houses where families live again, and take precautions, or family heads will choose to build or repair the drainage measures near the houses [47], so as to ensure that the houses can escape the disaster when the floods come. The impact of flood on the crops of farmers’ families is enormous, which makes the families whose income is agriculture lose their economic resources. Families will choose to increase and improve agricultural irrigation measures to alleviate the impact of flood on crops and minimize the possible impact of flood [48]. Based on the analysis of the occurrence time of perennial floods and the prediction results of floods, the farmers in flood-prone areas will change the types of crops into waterlogging-resistant crops to keep their crops able to withstand the impact of floods, or choose to advance or postpone the planting date of crops to avoid the invasion of floods, and ensure the family’s economic benefits by changing crops. Because of the perennial nature and great destructiveness of floods, families suffering from disasters choose to buy some natural disaster accident insurance and rely on insurance premiums to make up for the losses caused by floods [49]. This is a livelihood strategy choice of scientific disaster prevention. The destructive and destructive nature of floods has a great impact on farmers. Farmers can improve their awareness of disaster prevention and self-protection skills by participating in flood training [50]. Choosing this long-term livelihood prevention strategy can reduce losses. If suffering from long-term flooding, some families simply abandon their original homes and rebuild their homes on high ground that will not be invaded by flooding [51], so as to fundamentally solve the possible flooding problem, but the cost of such a choice is enormous.

### 3.2. Model Specification

Multinominal Logit Model (MNL) is often used in the study of the behavior choice [52,53,54,55]. This model can calculate the probability of farmers’ families choosing different livelihood strategies through a utility function. This study takes individual rural residents as the object and explores the influence of livelihood capital on rural residents’ choice of different livelihood strategies by establishing an MNL model.

Suppose that the nth respondent chooses the effect of the ith disaster preparedness behavior as U_ni_, J_n_ is the scheme set, then i ∈ J_n_, U_ni_ = V_ni_ + *ε*_ni_, and V_ni_ = *β*^′^X_nk_. Among them, *ε*_ni_ is the random error term; X_nk_ is the K factor which affects the nth disaster preparedness behavior; *β*^′^ is the parameter to be estimated. Then the probability that the nth respondent chooses the ith disaster preparedness behavior is:

(1) $$\begin{array}{l} P_{n}(i)=\mathrm{Prob}\left(U_{\mathrm{ni}}\geq U_{\mathrm{nj}},j\in J_{n},i\neq j\right)=\mathrm{Prob}\left(V_{\mathrm{ni}}+\varepsilon_{\mathrm{ni}},j\in J_{n},i\neq j\right)\\ =\mathrm{Prob}\left[V_{\mathrm{ni}}+\varepsilon_{\mathrm{ni}}\geq \underset{j\in J_{n}}{\max\left(V_{\mathrm{nj}}+\varepsilon_{\mathrm{nj}}\right)}\right] \end{array}$$

If each random term $\varepsilon_{\mathrm{ni}}$ obeys independent identical distribution, then $f(\varepsilon_{1},\varepsilon_{2},\ldots ,\varepsilon_{n})=\prod_{n}g(\varepsilon_{n})$ Where $g\left(\varepsilon_{n}\right)$ is the distribution function corresponding to the nth respondent. Assuming that $g\left(\varepsilon_{n}\right)$ obeys the double exponential distribution, the probability of choosing the ith disaster preparedness behavior in J_n_ is:

(2) $$p_{\mathrm{in}}=\frac{\exp\left(V_{\mathrm{in}}\right)}{\Sigma_{j\in J_{n}}\exp\left(V_{\mathrm{jn}}\right)}=\frac{1}{\Sigma_{j\in J_{n}}\exp\left(V_{\mathrm{jn}}\right)-\Sigma_{j\in J_{n}}\exp\left(V_{\mathrm{in}}\right)}=\frac{\exp\left(\beta^{\prime }X_{\mathrm{nk}}\right)}{\Sigma_{j\in J_{n}}\exp\left(\beta^{\prime }X_{\mathrm{nk}}\right)}$$

### 3.3. Sample Selection and Data Collection

**Figure 1:**
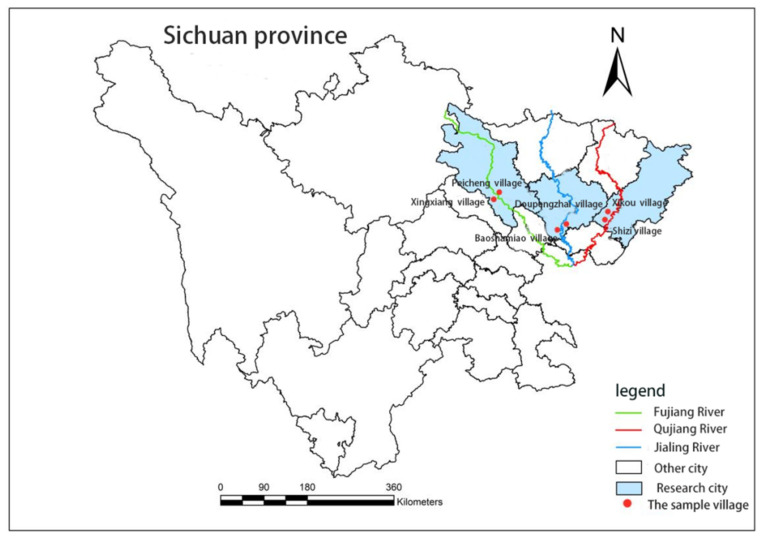
Geographical distribution of sample villages.

Based on the daily precipitation records of Sichuan Province from 1961 to 2017, it is known that Sichuan Province has experienced significant climate change [56,57]. Mianyang, Dazhou and Nanchong in eastern Sichuan belong to high-risk areas, high-sensitivity areas and high-vulnerability areas to storms and floods, and suffer from floods all year round [58]. Based on the research purpose and the relevant literature, it is found that the research sample points of flood disaster are basically communities along rivers or low-lying areas [59]. Therefore, when selecting sample villages, the villages with frequent floods along rivers are selected as sample points. Six sample villages are determined, namely Pengjiaxiang Village and Fucheng Village in Fujiang River Basin of Mianyang City, Baoshamiao Village and Diankouzhai Village in Jialing River Basin of Nanchong City, Shizi Village and Xikou Village in Qujiang River Basin of Dazhou City. The geographical distribution of the sample villages is shown in Figure 1.

Questionnaire data collection of influencing factors of household livelihood strategy choice in flood-prone areas is mainly divided into two stages: preparation and formal implementation. In the research preparation stage, the team recruited students from rural areas for the Chengdu University of Technology as the researchers and explained the research purpose, research methods, research contents, itinerary planning and time arrangement face to face. After all the preparatory work is completed, the research team is divided into three groups, taking the villagers’ activity center as the starting point, to visit the villagers in the sample village and random villagers are selected to conduct household survey, so as to ensure that the interviewees are evenly distributed in the village and do not repeat. Under the condition of certain error and confidence, the sample size will change with the overall size. The larger the total, the less obvious the change, while the smaller the total, the obvious change, but the change between them is not linear. Therefore, the larger the sample size, the better. The commonly used formula for calculating the minimum sample size is shown in (3), so the minimum sample size should be 269 when the confidence interval is 90% and the sampling error is 0.05.

(3) $$n=\frac{z^{2}\sigma^{2}}{d^{2}}$$

*n*: sample size
z: standard score of confidence interval; the value of z of 90% confidence interval is 1.64
*σ*: population standard deviation, and generally 0.5
*d*: sample error

The number of households in each village sampling survey is determined according to Formula (4) [60], to determine the minimum number of sampling copies. The sampling number of sample villages is shown in Table 2. A total of 360 questionnaires were distributed this time, and 325 questionnaires were recovered, with an effective recovery rate of 90.28%. It shows that the questionnaires collected in this survey meet the minimum sampling requirements, and further research can be carried out.

(4) $$n=\frac{N}{1+Ne^{2}}$$

*n*: number of households to be investigated in the sample village
*N*: the total number of households in the sample village
*e*: accuracy is set to 15% (0.15)

## 4. Results and Discussion

### 4.1. Farmers’ Household Short-Term Coping Strategy Choice Analysis

**Figure 2:**
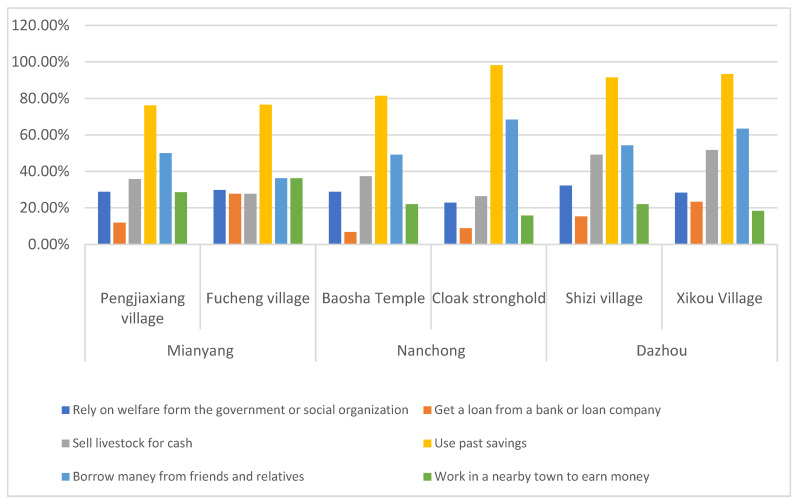
Proportion of farmer households in sample villages choosing livelihood strategies for flood disaster response.

According to the data of the household survey in the village, the proportion of farmer households in sample villages choosing livelihood strategies for flood disaster response is shown in Figure 2. Flood response strategies have the most options to use past savings. Pengjiaxiang village and Fucheng village in the Fujiang River Basin of Mianyang account for 76.19% and 76.60% respectively. In the Jialing River Basin of Nanchong, 81.36% of Baosha Temple village and 98.25% of Cloak Stronghold village. Shizi village and Xikou village in Dazhou Qujiang River Basin account for 91.53% and 93.33% respectively. It can be seen from this that most farmers have a certain amount of capital reserves to cope with the emergency needs of floods, however, in the short-term capital strategy to deal with flood disasters, the probability of choosing to borrow from banks or loan companies is low, which is due to the shortage of financial capital stock of farmers in the sample area.

**Figure 3:**
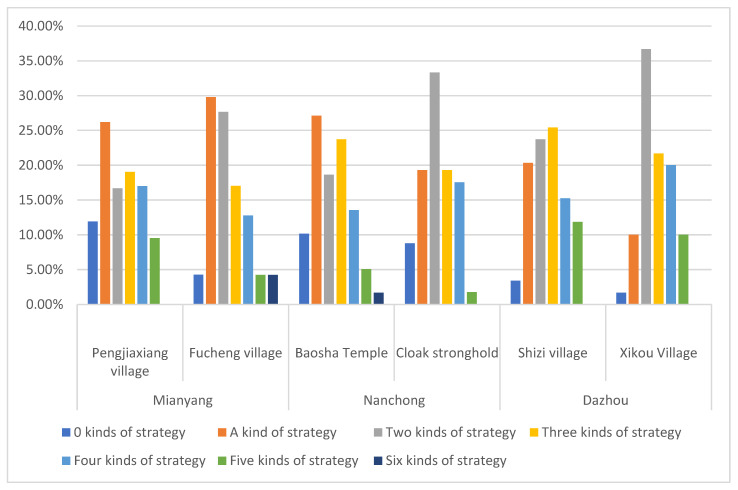
Proportion of types of flood disaster response strategies selected by farmer households in sample villages.

The proportions of farmer households choosing flood disaster response strategies is shown in Figure 3. 8.75% of them chose 0, that is, only a few families did not take corresponding financial strategies to deal with the flood risk when the flood came, the proportions of adopting 1, 2, 3, 4, 5 and 6 coping strategies were 21.60%, 26.54%, 21.30%, 16.05%, 7.1% and 1.00% respectively. Farmers choose 1–4 coping strategies when dealing with flood risk, accounting for 83.15% of farmers in the Mianyang Fujiang area, 86.21% in the Nanchong Jialing river area and 86.55% in the Dazhou Qujiang river area. It shows that most farmers in the study area will choose a variety of strategies to deal with the short-term financial difficulties when the flood comes, and families also have corresponding living capital storage to provide conditions for implementing the corresponding strategies.

### 4.2. Farmers’ Household Long-Term Adaption Strategy Choice Analysis

**Figure 4:**
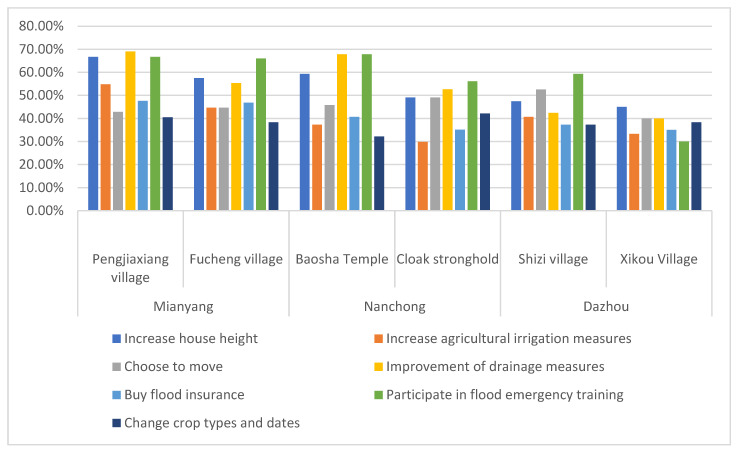
Proportion of households in sample villages choosing flood disaster adaptation strategy.

The proportion of farmer households choosing flood disaster adaptation livelihood strategy is shown in Figure 4, and the most selected strategy to adapt to floods is to participate in flood emergency training. Pengjiaxiang village and Fucheng village in the Fujiang River Basin of Mianyang account for 66.67% and 65.96% respectively. In the Jialing River Basin of Nanchong, 67.80% of Baosha Temple village and 56.14% of Cloak Stronghold village. Shizi village and Xikou village in Dazhou Qujiang River Basin account for 59.32% and 30% respectively. It can be seen that most farmer families in Jialing River Basin have been invaded by floods for a long time, and families will choose corresponding community flood emergency training to learn more emergency knowledge of flood escape or property protection, so as to reduce the losses caused by disasters. However, among all the strategies, the probability of choosing changing crop types and planting dates as the strategy to adapt to floods is low. This is because farmers’ habitual planting of crops and planting time has been deeply rooted in the sample area. Although farmers’ habits from ancient times to today face the challenge of flood, most families will still solve the farming problems caused eventually [61].

**Figure 5:**
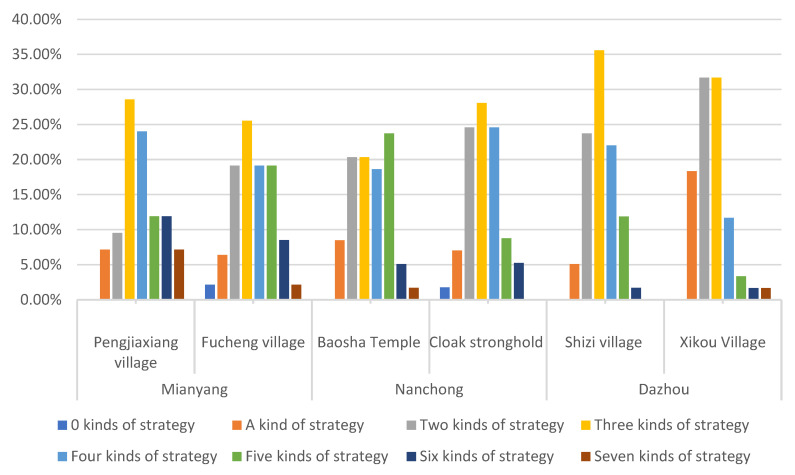
Proportion of types of flood disaster adaptation strategies selected by farmer households in sample villages.

The survey results reveal that farmers who choose no strategies to adapt to floods account for less than 1%. Only one family in Fucheng village in Mianyang and one family in Cloak Stronghold village in Nanchong did not adopt any flood adaptation strategy. It can be seen that only a very small number of farmers’ livelihood capital stock is at a low level, and the accumulated livelihood capital is small. The proportions of adopting 1, 2, 3, 4, 5, 6 and 7 flood adaptation strategies are 9%, 22.22%, 28.40%, 19.75%, 12.96%, 5.25% and 1.85% respectively. The proportion of farmers who choose 2–5 strategies is relatively high. The proportion of farmers in the Fujiang River area of Mianyang is 78.65%, that in the Jialing River area of Nanchong is 84.48%, and that in Qujiang River of Dazhou is 85.71%. Most farmers in the study area will choose a variety of livelihood strategies to adapt to the perennial floods, and families also have corresponding livelihood capital storage to provide conditions for implementing adaptation strategies. The number of livelihood strategies types selected by farmers is shown in Figure 5.

### 4.3. Household Livelihood Capital Impact on the Choice of Flood Response and Adaptation Strategies 

Using SPSS23.0 software, the Multinominal Logit Model model (MNL) was established to study the influence of farmer household livelihood strategy choice behavior and adaptation livelihood strategy choice behavior in flood-prone areas of Jialing River Basin. The proportion of coping flood without any livelihood strategy behavior is 8.75%, and this part of the questionnaire is screened out. The coping with the livelihood strategy behavior analysis uses the past savings as the reference group, and model fitting significance *p* is 0.000 (<0.050) and Nagellkerke R² is 0.378. The proportion of adaptation strategy model without any strategy is less than 1%, so this part of the questionnaire is screened out. The selection of perfect drainage measures is set as the reference group in adaptation strategy, and the model fitting significance *p* is 0.000 (<0.050) and Nagellkerke R² is 0.443. It can be judged that the fitting effect of the model is good, and the fitting results of the MNL model are shown in Table 3.

According to the fitting results of the MNL model, the choice behavior of farmer households’ livelihood strategy in flood-prone areas is affected by livelihood capital, and households with sufficient livelihood capital reserves have a stronger ability to cope with and adapt to flood risks. 

In human capital, compared with the coping strategy of families using their past savings to solve the lack of livelihood during the flood, the choice of family heads to work and earn money in nearby areas without disaster (*p* = 0.007, B = −1.755), relying on government or social organizations’ relief funds (*p* = 0.004, B = −0.325) and loans from banks or loan companies (*p* = 0.041, B = −1.081) is significantly negatively correlated with age, while the choice of changing sellers to produce livestock for cash (*p* = 0.005, B = 0.544) is significantly positively correlated with age, which is consistent with the conclusion of Coninx [46] on the choice of coping strategies of farmers’ families when the flood disaster comes. In addition, it also shows that after the disaster, older farmers are more inclined to choose to rely on themselves than the government or banks. Borrowing from banks or loan companies (*p* = 0.034, B = 2.150) is significantly positively correlated with the education level of household heads, which is consistent with the research conclusion of Bhattacharjee [28]. Compared with the choice of flood adaptation livelihood strategy to repair drainage facilities, the education level of the household head has a significant positive impact on the increase of house height (*p* = 0.001, B = 0.036), improvement of agricultural irrigation measures (*p* = 0.002, B = 0.162) and purchase of flood insurance (*p* = 0.000, B = 0.110), which is consistent with the research conclusion of Bhattacharjee [28].

In physical capital, compared with the coping strategy of families using their past savings to solve the lack of livelihood during the flood, the choice behavior of selling family property or livestock for cash (*p* = 0.023, B = −2.133) is negatively correlated with the construction area of the family house, which is consistent with the research conclusion of Xun [47]. Compared with choosing to repair drainage facilities in the flood livelihood strategy, buying flood protection (*p* = 0.034, B = 0.380), participating in flood emergency training (*p* = 0.030, B = 0.562), increasing agricultural irrigation measures (*p* = 0.047, B = 0.579), and changing crop planting date or planting (*p* = 0.004, B = 1.023) are positively correlated with the building area of family houses. Yang [48] also believes that when the housing construction area of farmer households is larger, they will have sufficient funds to choose more livelihood strategies. However, choosing to move (*p* = 0.003, B = −0.198) is negatively related to the building area of family houses, because the cost of implementing this strategy will be higher.

In natural capital, compared with using past savings, the exchange of property for cash (*p* = 0.000, B = −2.199) is significantly negatively correlated with the distance between the house and the river and lake, which is consistent with the research conclusion of Baker [49]. The closer the distance to the river, the greater the loss and damage caused by the flood, and selling the property can quickly solve the economic needs. In addition, farmers with drainage ditches are more likely to choose to rely on government or social organizations for relief funds (*p* = 0.001, B = 0.817), borrowing from banks or loan companies (*p* = 0.03, B = 0.405), selling property and livestock in exchange for cash (*p* = 0.005, B = 0.103). Compared with the adaptive livelihood strategy of improving drainage facilities, the choice of increasing house height (*p* = 0.001, B = −0.454) and moving (*p* = 0.004, B = −0.634) are significantly negatively correlated with the distance from the house to the river and lake, which is consistent with the research conclusions of Farman et al. [50] and Gioli et al. [51]. Farmers with drainage ditches are more likely to choose to increase agricultural irrigation measures (*p* = 0.00, B = 0.087), and farmers with drainage ditches will choose a variety of flood disaster response livelihood strategies, but the choice of flood disaster adaptation livelihood strategies is limited.

In financial capital, compared with using past savings to solve the lack of livelihood during the flood, there is a significant positive correlation between credit opportunities and borrowing from banks or loan companies (*p* = 0.001, B = 1.147), which can be explained by the fact that households have a loan history, indicating that they have a good understanding of the bank’s model and other conditions, and can also be able to do so when disasters occur. Guo [52] also draws consistent research conclusions. Compared with the livelihood adaptation strategy of choosing improved drainage facilities, the average annual household income has the greatest positive impact on increasing the height of the house (*p* = 0.031, B = 0.761). Rehman [53] also showed that family income was significantly positively correlated with the adaptive strategies made by families.

In social capital, compared with using past savings to solve the lack of livelihood during the flood, employment in villages has a positive impact on relying on the government or social company relief (*p* = 0.000, B = 0.817), which is consistent with the study of Bhattacharjee [32]. Compared with choosing the adaptive livelihood strategy of improving drainage facilities, having family members as cadres in the village and community has the greatest positive impact on choosing to participate in flood emergency training (*p* = 0.032, B = 0.505), followed by choosing to buy flood insurance (*p* = 0.018, B = 0.257). Wu et al. [42] in the research on the influencing factors of herdsmen’s livelihood capital on livelihood strategies also confirmed that the family’s employment as village cadres has a significant impact on the choice of herdsmen’s livelihood strategies.

## 5. Conclusions

Facing the climate change challenges, short-term and long-term livelihood strategies are of great importance to help farmers to be resilient in disaster-prone areas. Previous research only study the impact of various livelihood capital on disaster preparedness and post disaster recovery when farmers respond to disasters. The purpose of this study is to subdivide each livelihood capital into sub capitals and analyze their impacts on both short and long-term livelihood strategies. We aim to provide suggestions based on the analysis accordingly. This study applies the Department for International Development (DFID) farmer household sustainable livelihood analysis framework to conduct field investigation of household surveys in six sample villages of Dazhou Qujiang, Mianyang Fujiang and Nanchong Jialing River in Sichuan. Based on the analysis of the current situation of household livelihood capital, the factors affecting the household’s choice of livelihood strategies to cope with floods and adapt to floods, this study constructs the household livelihood capital evaluation index system. Using the collected data and Multinominal Logit Model (MNL) analysis, the research draws the following conclusions: Through investigation and analysis, it can be seen that most of the farmer households in the flood-prone areas in the six sample villages have a certain amount of capital reserves to deal with flood disasters, but the proportion of responding to disasters by borrowing from banks or loan companies is relatively low, And farmer households generally choose 1–4 strategies to deal with floods. Among the livelihood strategies that farmers choose to adapt to flood disasters, participating in flood emergency training accounted for the largest proportion of the sample villages, while the proportion of choosing to change the type and date of crop planting was the least. The coping strategies adopted by farmers when adapting to flood risks with 2–5 species as the main type, and less than 1% of farmers in flood-prone areas did not choose any flood-adaptive livelihood strategies. According to the fitting results of the MNL model, the choice behavior of farmers’ livelihood strategy in flood-prone areas is affected by the status of farmers’ livelihood capital. Farmers’ families with sufficient reserves of livelihood capital have a stronger ability to cope with and adapt to flood risk.

In terms of human capital, farmers with higher education can choose more scientific and effective livelihood strategies to deal with and adapt to floods, however, the choice of livelihood strategies for older farmers is limited. Therefore, local governments should increase local investment in education to enable farmers and families to have more choices of livelihood strategies to deal with and adapt to floods. At the same time, banks should appropriately relax the age limit for applying for loans to the elderly who meet the credit conditions, so that the elderly can receive financial assistance in the event of disasters.

Considering physical capital, households with larger built-up areas tend to adopt rich adaptation strategies (increasing agricultural irrigation, changing crop planting types and dates, purchasing flood insurance, participating in flood emergency training), when the housing area of farmers’ families is larger, their economy is richer and they have more funds to make livelihood strategies to adapt to floods. We can increase the material capital by increasing family fixed assets, appropriate compensation from the government, improving housing conditions and increasing the source of income of farmers. At the same time, we can strengthen the demolition and reconstruction of old and dilapidated houses in this area and improve the ability of houses to cope with the impact of floods.

Talking about natural capital, families closer to rivers and lakes will choose measures that can be quickly realized (selling livestock, working), while families with drainage ditches will choose richer flood response strategies. it is needed to improve the grass-roots transportation network and the family location advantages by increasing the cultivated land area of farmers’ families. The output value of cultivated land per hectare and land-use efficiency should be improved. We need to intensify efforts to build flood control and waterlogging prevention measures, and strengthen the construction of rural drainage facilities and flood control dams for rivers.

For financial capital, farmers and families with more credit opportunities are more willing to choose loans to deal with floods. Improving the microfinance system to facilitate farmers’ family loans is therefore necessary. At the same time, we should improve the employment rate of the rural population and increase the average annual income of farmers’ families. Sufficient financial capital reserves can enable households to have more and more effective livelihood strategies to choose in the process of flood response and adaptation.

Regarding social capital, with the help of the village or neighbors for a long time, farmers’ families will not need to choose to move to adapt to the flood disaster. They can increase the publicity and support of farmers’ cooperative organizations, integrate village social relationship network resources, and enhance the collective ability to resist the flood risk. Farmers and families shall be encouraged to strengthen contact with relatives and friends. Relevant local government departments shall timely and truly inform local farmers of the real-time flood information and the flood control measures taken, so as to enhance the trust of local farmers in the flood control capacity of the media and the government.

This study also plays an important role in other parts of the world. It expands the research area on the impact of both short-term and long-term flood-response strategies and adaptation strategies of farmers under different sub-divided livelihood capitals, and enriches therefore the theoretical analysis framework of livelihood capital and livelihood strategies in disaster-prone areas. Other areas can follow the same analysis framework to customize their analysis. It also provides theoretical reference for further research and analysis on sustainable development of farm households in similar regions.

This research has several limitations that should be addressed in the future research. On the one hand, the selected areas are only the flood-prone rural areas in Sichuan. Whether they are representative for the whole country needs further investigation in flood-prone rural areas in other provinces. On the other hand, although the sample size meets the minimum sample requirements, obtaining more samples will make the data analysis more accurate. Last but not least, although several rural areas in different regions were selected, samples from different regions were not compared, so it is necessary to further explore whether there is spatial heterogeneity in each variable in future studies.

## Figures and Tables

**Table 2:** Sample number of sample villages.

Sample Villages		Total Households	Number of Sample Households	Number of Valid Survey	Sampling Rate
Fujiang River Basin of Mianyang City	Pengjiaxiang Village	726	42	42	5.79%
	Fucheng Village	1155	46	43	4.36%
Jialing River Basin of Nanchong City	Cloak Stronghold	854	58	42	6.79%
	Baosha Temple	483	59	41	12.21%
Qujiang River Basin of Dazhou City	Xikou Village	760	60	43	7.89%
	Shizi Village	1080	60	42	5.55%

**Table 3:** MNL model parameter estimation.

MNL Model Fitting Results of Farmers’ Household Livelihood Capital on the Choice of Livelihood Strategies in Response to Floods											MNL Model Fitting Results of Farmers’ Household Livelihood Capital on the Choice of Livelihood Strategies in Flood Disaster Adaptation											
Index	Rely on Welfare from the Government or Social Organization		Get a Loan from a Bank or Loan Company		Sell Livestock for Cash		Borrow Money from Friends and Relatives		Work in a Nearby Town to Earn Money		Increase House Height		Choose to Move		Increase Agricultural Irrigation Measures		Change Crop Types and Dates		Buy Flood Insurance		Participate in Flood Emergency Training	
	B	*p*-Value	B	*p*-Value	B	*p*-Value	B	*p*-Value	B	*p*-Value	B	*p*-Value	B	*p*-Value	B	*p*-Value	B	*p*-Value	B	*p*-Value	B	*p*-Value
intercept	−0.369	0.716	−1.636	0.167	−1.45	0.116	−1.121	0.183	−1.631	0.171	0.109	0.001	−0.138	0	0.143	0.001	−0.066	0.003	0.149	0.002	0.054	0
Human capital											Human capital											
Age of head of household	−0.325	0.004	−1.081	0.041	0.544	0.005	0.162	0.71	−1.775	0.007	−0.055	0.839	0.057	0.847	−0.159	0.545	−0.243	0.4	−0.111	0.636	−0.117	0.633
Education level of head of household	0.199	0.824	2.15	0.034	−0.407	0.616	0.217	0.768	1.952	0.044	0.036	0.001	0.142	0.205	0.162	0.002	0.107	0.013	0.118	0	0.11	0.021
Family illness	1.882	0.039	−0.534	0.022	0.126	0.562	0.147	0.456	−0.395	0.15	−0.056	0.813	−0.272	0.015	−0.221	0.298	−0.321	0.207	0.258	0.017	−0.241	0.235
Total family size	1.842	0.037	−1.325	0.035	0.337	0.537	−0.018	0.972	−0.651	0.35	−0.019	0.924	−0.226	0.304	−0.199	0.28	−0.011	0.957	−0.117	0.485	−0.134	0.44
Natural capital											Natural capital											
Own land area	−0.502	0.393	0.24	0.718	−0.029	0.953	−0.06	0.893	−0.384	0.549	−0.193	0.22	−0.15	0.01	0.332	0.005	0.176	0.002	−0.063	0.619	−0.091	0.499
Family location (the distance between the house and the river)	−0.601	0.014	−0.769	0.199	−2.199	0	−0.007	0.986	−1.877	0.002	−0.454	0.001	−0.634	0.004	0.186	0.687	0.311	0.564	−0.184	0.013	0.597	0.171
Drain condition	0.817	0.001	0.405	0.03	0.103	0.005	−0.238	0.256	−0.336	0.267	−0.144	0.664	−0.18	0.024	0.087	0	−0.011	0.977	−0.055	0.852	−0.019	0.949
Physical capital											Physical capital											
House area	−0.246	0.761	−1.022	0.267	−2.133	0.023	−0.509	0.423	−0.741	0.406	0.327	0.326	−0.198	0.003	0.579	0.047	1.023	0.004	0.38	0.034	0.562	0.03
House age	−0.015	0.98	−0.54	0.456	1.63	0.017	−0.187	0.707	0.932	0.169	0.08	0.016	−0.043	0.62	0.071	0.292	0.038	0.622	0.078	0.206	0.045	0.481
House structure	−0.246	0.761	−1.022	0.267	1.03	0	−0.509	0.423	−0.741	0.406	−0.058	0.041	0.281	0.11	0.052	0.73	0.188	0.264	0.145	0.297	0.148	0.299
Household livestock value	−0.03	0.935	−0.597	0.184	0.304	0.337	0.179	0.544	−0.245	0.568	−0.034	0.868	−0.056	0.801	0.06	0.05	0.195	0.014	−0.003	0.988	−0.09	0.62
Value of household items	0.05	0.959	−0.009	0.993	0.621	0.473	−0.113	0.891	−1.519	0.189	−0.162	0.34	−0.22	0.228	−0.12	0.457	0.133	0.447	0.037	0.015	−0.058	0.7
Financial capital											Financial capita											
Average annual household income	−0.814	0.168	−0.423	0.514	−0.476	0.355	−0.211	0.645	0.278	0.65	0.761	0.031	0.208	0.279	0.056	0.012	0.233	0.081	0.655	0.005	0.131	0.141
Average annual household income	0.583	0.353	0.356	0.622	0.025	0.963	0.229	0.64	−0.224	0.755	−0.204	0.554	0.685	0.052	−0.124	0.706	0.172	0.625	0.52	0.003	−0.37	0.016
Credit opportunity	0.031	0.928	1.147	0.001	0.072	0.81	0.367	0.162	0.726	0.037	−0.279	0.577	1.249	0.018	−0.015	0.974	−0.169	0.76	0.355	0.014	−0.02	0.962
Borrowing opportunity	0.208	0.437	0.033	0.913	0.262	0.272	0.671	0.003	0.275	0.357	0.197	0.649	−0.405	0.415	−0.048	0.908	0.109	0.818	−0.101	0.788	0.555	0.163
Social capital											Social capital											
Community help during disasters	0.458	0.016	0.308	0.134	0.712	0	0.519	0.003	0.805	0	−0.251	0.67	−0.547	0.032	−0.049	0.929	1.105	0.057	−0.006	0.991	0.062	0.001
Helped by neighbors during disasters	−0.079	0.788	0.552	0.141	−0.114	0.668	−0.298	0.219	−0.064	0.843	−0.095	0.831	−0.448	0.014	0.653	0.131	−0.496	0.303	−0.265	0.486	−0.524	0.186
Trust in village managers	−0.157	0.591	0.225	0.518	0.143	0.599	0.297	0.241	0.272	0.427	0.111	0.843	−0.085	0	−0.059	0.919	−0.053	0.93	0.24	0.016	−0.117	0.827
Occupation in the village group	0.817	0	0.405	0.018	0.229	0.247	−0.146	0.455	−0.012	0.958	0.222	0.018	−0.209	0.746	0.557	0.292	0.157	0.81	0.257	0.018	0.505	0.032

## Data Availability

The data are available from the corresponding author upon reasonable request.

## References

[1] Mavhura E. (2017). Applying a systems-thinking approach to community resilience analysis using rural livelihoods: The case of Muzarabani district, Zimbabwe. Int. J. Disaster Risk Reduct..

[2] Atube F., Malinga G.M., Martine N., Okello D.M., Okello-Uma I. (2021). Determinants of smallholder farmers’ adaptation strategies to the effects of climate change: Evidence from northern Uganda. Agric. Food Secur..

[3] Haeffner M., Baggio J.A., Galvin K. (2018). Investigating environmental migration and other rural drought adaptation strategies in Baja California Sur, Mexico. Reg. Environ. Chang..

[4] Cai Z. (2010). Analysis on livelihood capital of peasant households in poverty-stricken villages in Wenchuan earthquake area. Chin. Rural. Econ..

[5] Zhuang T., Zhang H., Yang J. (2010). Study on the impact of natural disasters on rural poverty in Southwest China—Based on the analysis of 67 villages in 21 national poverty counties. Rural. Econ..

[6] Cao S., Wang Y., Duan F., Zhao W., Wang Z., Fang N. (2016). Coupling relationship between ecological environmental vulnerability and economic poverty in poor areas of China: An empirical analysis of 714 poor counties in contiguous poor areas. Chin. J. Appl. Ecol..

[7] Yang Y., Wang H., He W. (2016). Research on the mode of Targeted financial poverty alleviation in China under the “four-element structure”. Tibet. Financ..

[8] Ma C., Liu L., Yuan C., Ren G. (2018). The differentiation characteristics of farmers’ livelihood capital and its impact on livelihood strategy in rapid urbanization area: A case study of Qingpu District, Shanghai. Study Agric. Mod..

[9] Yang X. (2018). Study on the Relationship between Livelihood Capital and Livelihood Strategy of Farmers in Desertification Land Restricted Reserve: A Case Study of Six Counties in Hexi Corridor of Gansu Province.

[10] Su F., Pu X., Xu Z., Wang L. (2009). Study on the relationship between livelihood capital and livelihood Strategy—A case study of Ganzhou District in Zhangye City. China Popul. Resour. Environ..

[11] Zhao Y. (2017). Study on Sustainable Livelihood of Poverty Alleviation Migrants in Inhospitable Areas. Master’s Thesis.

[12] Zhou W.F., Guo S.L., Deng X., Xu D.D. (2021). Livelihood resilience and strategies of rural residents of earthquake-threatened areas in Sichuan Province, China. Nat. Hazards.

[13] Amir S., Saqib Z., Khan M.I., Ali A., Khan M.A., Bokhari S.A., Zaman ul H. (2020). Determinants of farmers’ adaptation to climate change in rain-fed agriculture of Pakistan. Arab. J. Geosci..

[14] Mentamo M., Geda N.R. (2016). Livelihood diversification under severe food insecurity scenario among smallholder farmers in Kadida Gamela District, Southern Ethiopia. Kontakt.

[15] Rahut D.B., Mottaleb K.A., Ali A. (2018). Rural Livelihood Diversification Strategies and Household Welfare in Bhutan. Eur. J. Dev. Res..

[16] Su Y., Deng W., Zhang J., Hriday Lal K., Paudel Khatiwada S. (2016). Relationship between Livelihood Capital and Livelihood Strategies of Rural Households in Melamchi Basin of Central Mountainous Area in Nepal. Mt. Res..

[17] Wei B., Su G., Li Y., Ma Y. (2019). Livelihood Strategies of Rural Households in Ning’er Earthquake-Stricken Areas, Yunnan Province, China. Sustainability.

[18] Qin Y., Shi X., Li X., Yan J. (2021). Geographical indication agricultural products, livelihood capital, and resilience to meteorological disasters: Evidence from kiwifruit farmers in China. Environ. Sci. Pollut. Res..

[19] Zhou W., Ma Z., Guo S., Deng X., Xu D. (2021). Livelihood capital, evacuation and relocation willingness of residents in earthquake-stricken areas of rural China. Saf. Sci..

[20] Shah A.A., Ye J., Abid M., Ullah R. (2000). Determinants of flood risk mitigation strategies at household level: A case of Khyber Pakhtunkhwa (KP) province, Pakistan. Nat. Hazards.

[21] Dalu M.T.B., Shackleton C.M. (2018). The potential use of natural resources in urban informal settlements as substitutes for financial capital during flooding emergencies. Phys. Chem. Earth.

[22] Memon M.H., Ali M., Khalil S. (2020). Determinants of income diversification in flood-prone rural Pakistan. Int. J. Disaster Risk Reduct..

[23] Collier B.L., Babich V.O. (2019). Financing Recovery After Disasters: Explaining Community Credit Market Responses to Severe Events. J. Risk Insur..

[24] Adelekan I.O., Asiyanbi A.P. (2016). Flood risk perception in flood-affected communities in Lagos, Nigeria. Nat. Hazards.

[25] Lee D.W., Kim H.Y. (2021). The effect of social capital on disaster conflicts in local communities: Focusing on disaster victims. Int. J. Disaster Risk Reduct..

[26] Sadri A.M., Ukkusuri S.V., Lee S., Clawson R., Aldrich D., Nelson M.S., Seipel J., Kelly D. (2018). The role of social capital, personal networks, and emergency responders in post-disaster recovery and resilience: A study of rural communities in Indiana. Nat. Hazards.

[27] Nakayachi K. (2015). Examining Public Trust in Risk-Managing Organizations After a Major Disaster. Risk Anal..

[28] Yang L., Ao Y., Ke J., Lu Y., Liang Y. (2021). To walk or not to walk? Examining non-linear effects of streetscape greenery on walking propensity of older adults. J. Transp. Geogr..

[29] Yang L., Chau K.W., Szeto W.Y., Cui X., Wang X. (2020). Accessibility to transit, by transit, and property prices: Spatially varying relationships. Transp. Res. Part D Transp. Environ..

[30] Yang L., Liang Y., He B., Lu Y., Gou Z. (2022). COVID-19 effects on property markets: The pandemic decreases the implicit price of metro accessibility. Tunn. Undergr. Space Technol..

[31] Yang L., Chu X., Gou Z., Yang H., Lu Y., Huang W. (2020). Accessibility and proximity effects of bus rapid transit on housing prices: Heterogeneity across price quantiles and space. J. Transp. Geogr..

[32] Bhattacharjee K., Behera B. (2018). Determinants of household vulnerability and adaptation to floods: Empirical evidence from the Indian State of West Bengal. Int. J. Disaster Risk Reduct..

[33] Kong L., Li Y., Wang M., Zheng T. (2021). Research on livelihood Capital and Livelihood Strategy of Peasant households under project poverty Alleviation—Based on investigation data of Shule County, Xinjiang. Agric. Resour. Reg. China.

[34] Saqib S.E., Ahmad M.M., Panezai S., Rana I.A. (2016). An empirical assessment of farmers’ risk attitudes in flood-prone areas of Pakistan. Int. J. Disaster Risk Reduct..

[35] Zhao X. (2011). Impact of livelihood Capital on life satisfaction of Farmers and herdsmen: A case study of Gannan Plateau. Geogr. Res..

[36] Hao W., Yang D., Zhang J., Li W., Wang Z. (2014). A study on the relationship between sustainable livelihood Capital and livelihood Strategy of Farmers and Herdsmen: A case study of Nyingchi, Tibet. J. Arid. Land Resour. Environ..

[37] Wang L., Wang C., Li X. (2012). Household differentiation based on livelihood asset quantification: A case study of 471 households in Bailin Village, Shapingba District, Chongqing. Geogr. Res..

[38] Danso S.Y., Addo I.Y. (2016). Coping strategies of households affected by flooding: A case study of Sekondi-Takoradi Metropolis in Ghana. Urban Water J..

[39] Atreya A., Czajkowski J., Botzen W., Bustamante G., Campbell K., Collier B., Montgomery M. (2017). Adoption of flood preparedness actions: A household level study in rural communities in Tabasco, Mexico. Int. J. Disaster Risk Reduct..

[40] Yuan Q. (2019). Study on Sustainable Livelihood of Farmers in Zhangjiajie under the Background of Rural Ecotourism Development. Master’s Thesis.

[41] Ma L. (2017). Study on Flood Vulnerability and Sustainable Livelihood Strategies of Farmers in Poyang Lake Region. Master’s Thesis.

[42] Wu T., Wu Y., Wang D., Lin H. (2019). Study on the impact of livelihood capital on livelihood strategy of herders in Sanjiangyuan Region. J. Grass Ind..

[43] Ao Y., Zhang H., Yang L., Wang Y., Martek I., Wang G. (2021). Impacts of earthquake knowledge and risk perception on earthquake preparedness of rural residents. Nat. Hazards.

[44] Liang Y., Cao R. (2015). Employment assistance policies of Chinese government play positive roles! The impact of post-earthquake employment assistance policies on the health-related quality of life of Chinese earthquake populations. Soc. Indic. Res..

[45] Mondal M.S.H., Murayama T., Nishikizawa S. (2021). Determinants of Household-Level Coping Strategies and Recoveries from Riverine Flood Disasters: Empirical Evidence from the Right Bank of Teesta River, Bangladesh. Climate.

[46] Helgeson J.F., Dietz S., Hochrainer-Stigler S. (2013). Vulnerability to Weather Disasters: The Choice of Coping Strategies in Rural Uganda. Ecol. Soc..

[47] Abebe Y.A., Ghorbani A., Nikolic I., Vojinovic Z., Sanchez A. (2019). Flood risk management in Sint Maarten—A coupled agent-based and flood modelling method. J. Environ. Manag..

[48] Schad I., Schmitter P., Saint-Macary C., Neef A., Lamers M., Nguyen L., Hilger T., Hoffmann V. (2012). Why do people not learn from flood disasters? Evidence from Vietnam’s northwestern mountains. Nat. Hazards.

[49] Bao X., Zhang F., Deng X., Xu D. (2021). Can Trust Motivate Farmers to Purchase Natural Disaster Insurance? Evidence from Earthquake-Stricken Areas of Sichuan, China. Agriculture.

[50] Xu D., Peng L., Liu S., Wang X. (2018). Influences of Risk Perception and Sense of Place on Landslide Disaster Preparedness in Southwestern China. Int. J. Disaster Risk Sci..

[51] King D., Bird D., Haynes K., Boon H., Cottrell A., Millar J., Okada T., Box P., Keogh D., Thomas M. (2014). Voluntary relocation as an adaptation strategy to extreme weather events. Int. J. Disaster Risk Reduct..

[52] Wang F., Ross C.L. (2018). Machine Learning Travel Mode Choices: Comparing the Performance of an Extreme Gradient Boosting Model with a Multinomial Logit Model. Transp. Res. Rec..

[53] Yang L. (2018). Modeling the mobility choices of older people in a transit-oriented city: Policy insights. Habitat Int..

[54] Guo Y., Yang L., Huang W., Guo Y. (2020). Traffic safety perception, attitude, and feeder mode choice of metro commute: Evidence from Shenzhen. Int. J. Environ. Res. Public Health.

[55] Ao Y., Zhang Y., Wang Y., Chen Y., Yang L. (2020). Influences of rural built environment on travel mode choice of rural residents: The case of rural Sichuan. J. Transp. Geogr..

[56] Li J., Zhao Y., Iqbal J. (2019). Variation patterns of extreme precipitation and relation to ocean-atmospheric climate in Sichuan province China from 1961 to 2017. Theor. Appl. Climatol..

[57] Chen C., Pang Y., Zhang Y. (2010). The characteristics of climate change in Sichuan Basin in recent 50 years. J. Southwest Univ..

[58] Du H., Dong T. (2016). Risk assessment of rainstorm and flood in main flood season in Sichuan-Yunnan region. Bull. Soil Water Conserv..

[59] Roy P., Pal S.C., Chakrabortty R., Chowdhuri I., Malik S., Das B. (2020). Threats of climate and land use change on future flood susceptibility. J. Clean. Prod..

[60] Yamane T. (1965). Statistics: An Introductory Analysis. Harper Row.

[61] Nguyen A.T., Hens L. (2021). Diversified responses to contemporary pressures on sloping agricultural land: Thai farmer’s perception of mountainous landscapes in northern Vietnam. Environ. Dev. Sustain..

